# Supplementary material for: SARS-CoV-2 Rapidly Infects Peripheral Sensory and Autonomic Neurons, Contributing to Central Nervous System Neuroinvasion before Viremia
Source: Int J Mol Sci. 2024 Jul 28;25(15):8245. doi: 10.3390/ijms25158245 (PMC11311394; doi:10.3390/ijms25158245)
Supplement: Supplementary file 1 [file ijms-25-08245-s001.zip › ijms-3045532-supplementary.pdf]

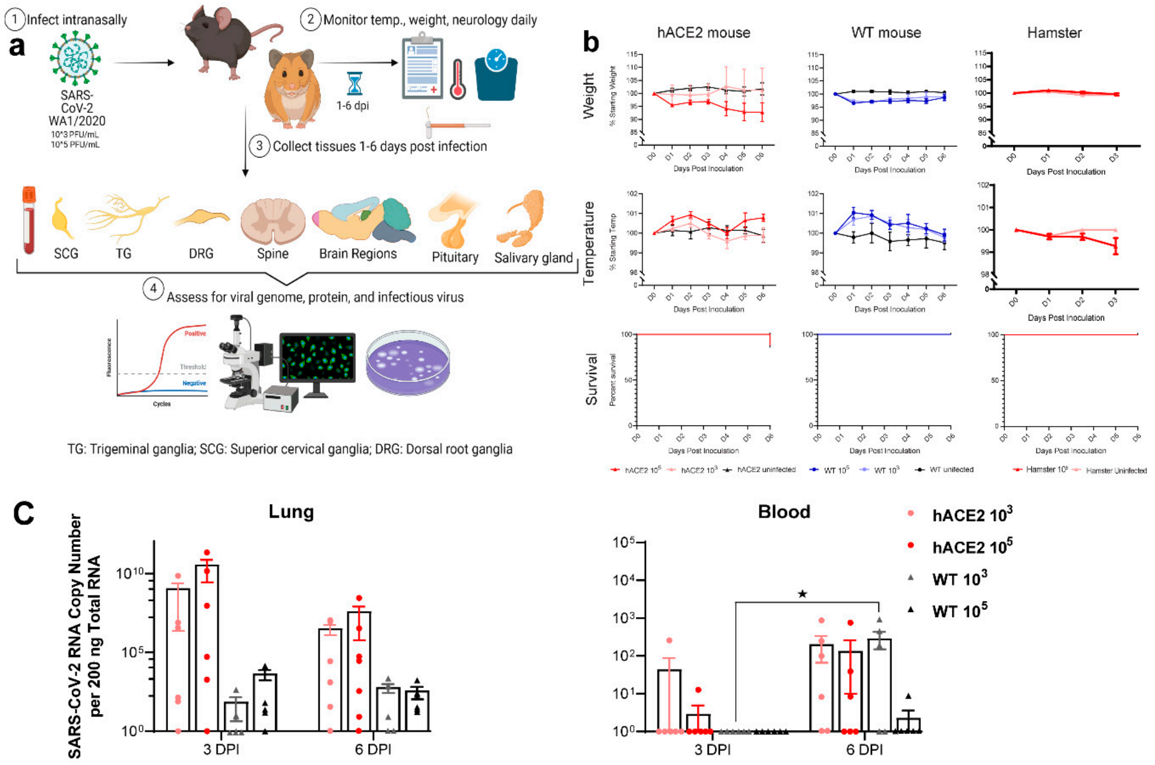

Supplementary Figure S1. Experimental approach and clinical data for hACE2 mice, WT mice, and golden Syrian hamsters. (**a**), Graphical abstract outlining the experimental approach used in mouse (hACE2: n=35; WT: n=34; controls: n=4/mouse type) and Syrian golden hamster (n=9; control: n=1) infections highlighting intranasal infection of groups with either 3 log PFU or 5 log PFU SARS-CoV-2 (mice) or 5 log PFU SARS-CoV-2 (hamsters), clinical evaluation (temperature, weight, survival, von Frey threshold), collection of tissues, and downstream analysis for SARS-CoV-2 RNA copies (RT-qPCR), virus and host antigen (immunostaining), and infectious virus (plaque assay). (**b**), Clinical data by inoculum group for mice and hamsters including weight, temperature, and survival. Weight (grams) for each animal was recorded daily and reported as the mean percentage increase/decrease (±SD) for each inoculum group relative to the mean starting weight for that group. The only group to exhibit notable weight loss was the hACE2 mice inoculated with 5 log PFU, which began to lose weight after 3 dpi. A minor but insignificant decrease in weight was observed in infected hamsters relative to uninfected control. Temperature (°C) for each animal was recorded daily. Temperature is reported as the mean percentage increase/decrease for each inoculum group relative to the mean starting temperature for that group. A transient temperature increases occurred in the 5 log PFU inoculated mice whereas a mild decrease occurred in 5 log PFU inoculated hamsters. Kaplan-Meier survival plots were created for each inoculum group. The only group to have mortality was the hACE2 mice inoculated with 5 log PFU. Mortality was noted at 6 dpi (14%, n= 2 of 14). (**c**), SARS-CoV-2 RNA was detected in lungs of hACE2 and WT mice in both inocula groups at both timepoints, which decreased over time. While differences were detected in the lungs ($F(7, 41) = 2.745$, $p = 0.0197$) none were between relevant groups. Low concentrations of SARS-CoV-2 RNA were detected in the blood of hACE2 in both inoculum groups at 3 dpi and both hACE2 and WT mice in both inoculum groups at 6 dpi. A significant difference ($F(7, 40) = 3.417$, $P = <0.006$) was detected in the WT group inoculated with 10^3 PFU assessed at 3- vs 6-dpi ($p= 0.0321$). Data are the mean ± s.e.m. Log transformed RNA genome copy numbers were statistically compared by three-way ANOVA (independent variables: inocula, days post infection, genotype). Pairwise comparisons were conducted using Tukey's HSD post hoc tests. *$p < 0.05$, **$p < 0.01$, ***$p < 0.001$. See Figure S6 for hACE2 genotyping, hACE2 protein expression, and RT-qPCR controls.

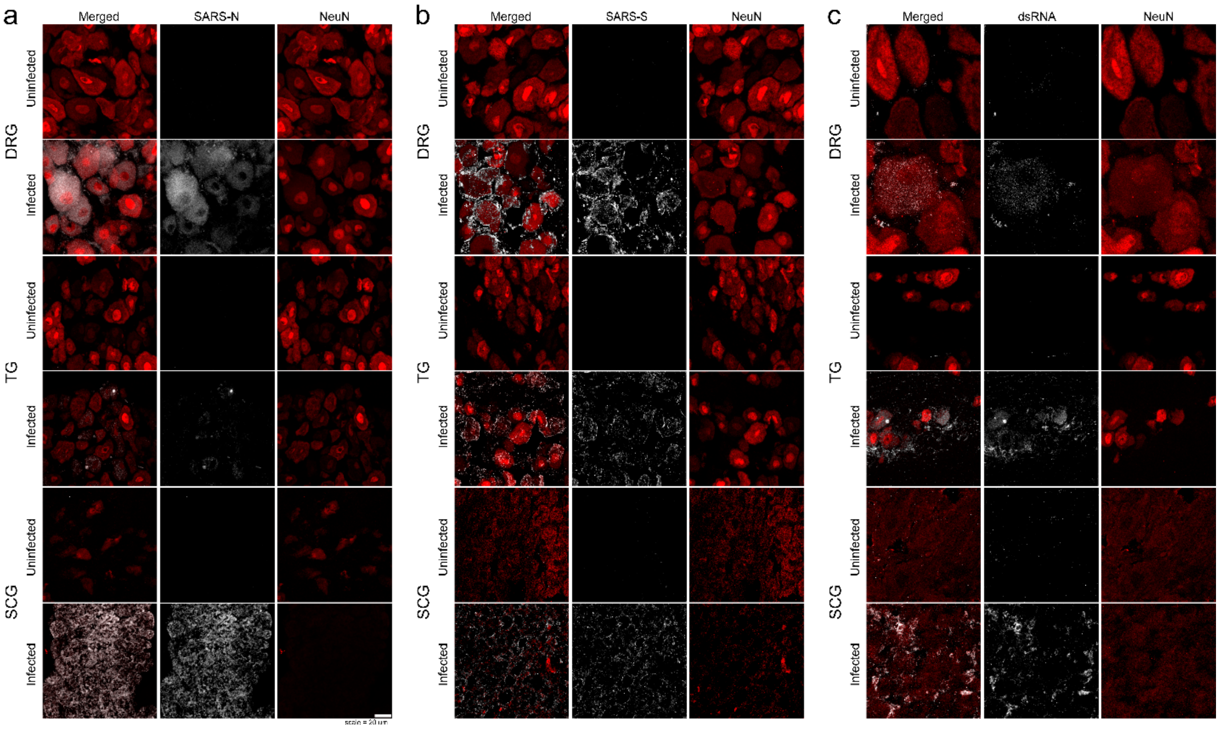

Supplementary Figure S2. Immunofluorescence for SARS-N protein, SARS-S protein, dsRNA, and NeuN in peripheral ganglia from hACE2 mice. All images were acquired using a Leica SP8 confocal microscope, using identical image acquisition settings (laser power and gain) across all sections shown for each SARS-related antibody. All images were colorized, z-projected, and prepared using identical contrast and brightness parameters in ImageJ for each SARS-related antibody. (**a**), SARS-N is present in neurons in the LS-DRG, TG and SCG in 10^5 PFU-inoculated mice. Minimal background immunofluorescence is observed in sections from uninfected mice. Of note is substantial vacuolization and loss of NeuN immunofluorescence in the infected SCG. (**b**), SARS-S is present in neurons in the LS-DRG, TG and SCG in 10^5 PFU-inoculated mice, whereas there is some extracellular nonspecific staining visible, likely due to mouse-on-mouse immunohistochemistry artifacts. (**c**), dsRNA is present in neurons and some satellite glial cells in the LS-DRG, TG and SCG in 10^5 PFU-inoculated mice. Similar to SARS-S, some nonspecific immunostaining is present, also likely due to mouse-on-mouse immunostaining artifacts. The difference between infected and uninfected cells is readily apparent, however, for SARS-S and dsRNA. See Figure S6 for additional antibody validation via western blot, hACE2 genotyping, and hACE2 protein expression.

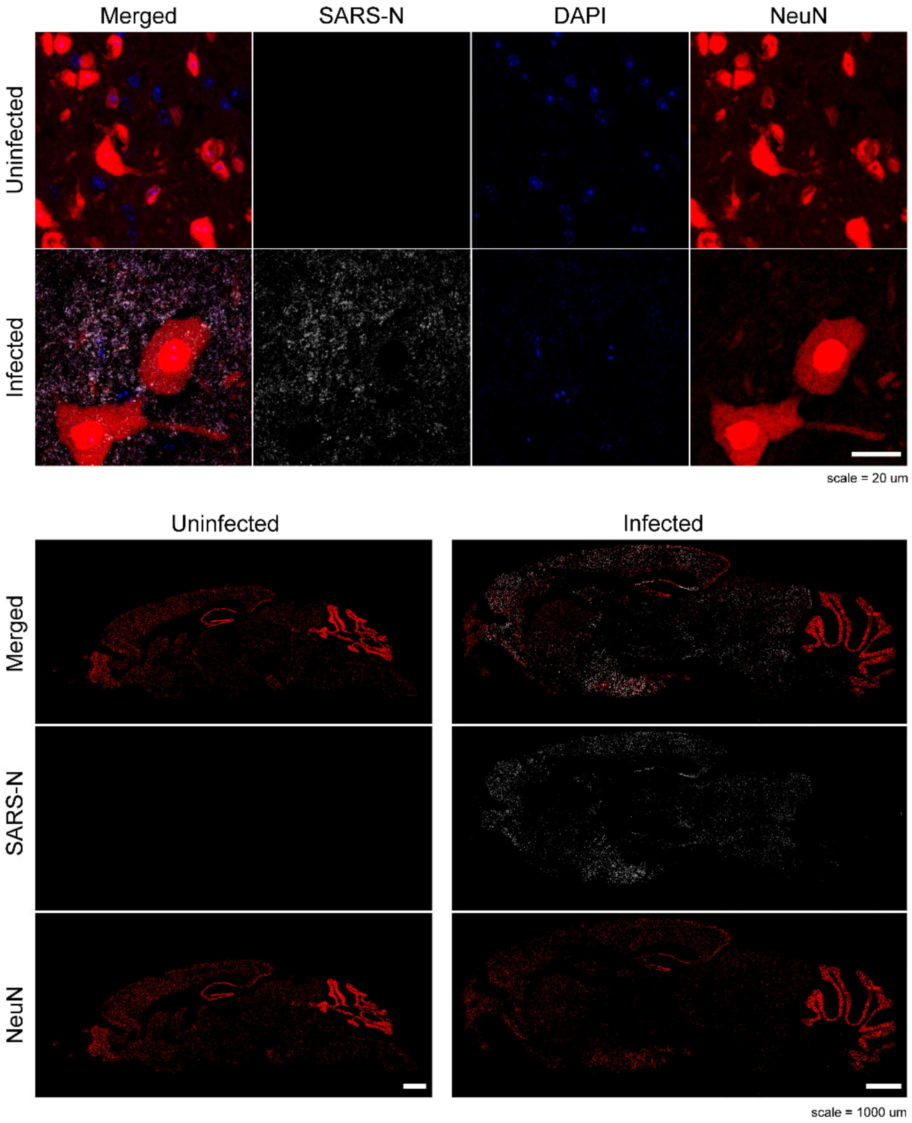

Supplementary Figure S3. Immunofluorescence for SARS-N protein and NeuN in sections of spinal cords and brains taken from infected and uninfected hACE2 mice. All images were acquired using a Leica SP8 confocal microscope, using identical image acquisition settings (laser power and gain) across all sections from each tissue type shown. All images were colorized, z-projected, and prepared using identical contrast and brightness parameters in ImageJ. SARS-N is present in neurons in the spinal cord and brains in infected mice. Minimal background immunofluorescence is observed in sections from uninfected mice. Of note, SARS-N intensity was qualitatively substantially higher in the brain than in the spinal cord. See Figure S6 for additional antibody validation via western blot, hACE2 genotyping, and hACE2 protein expression.

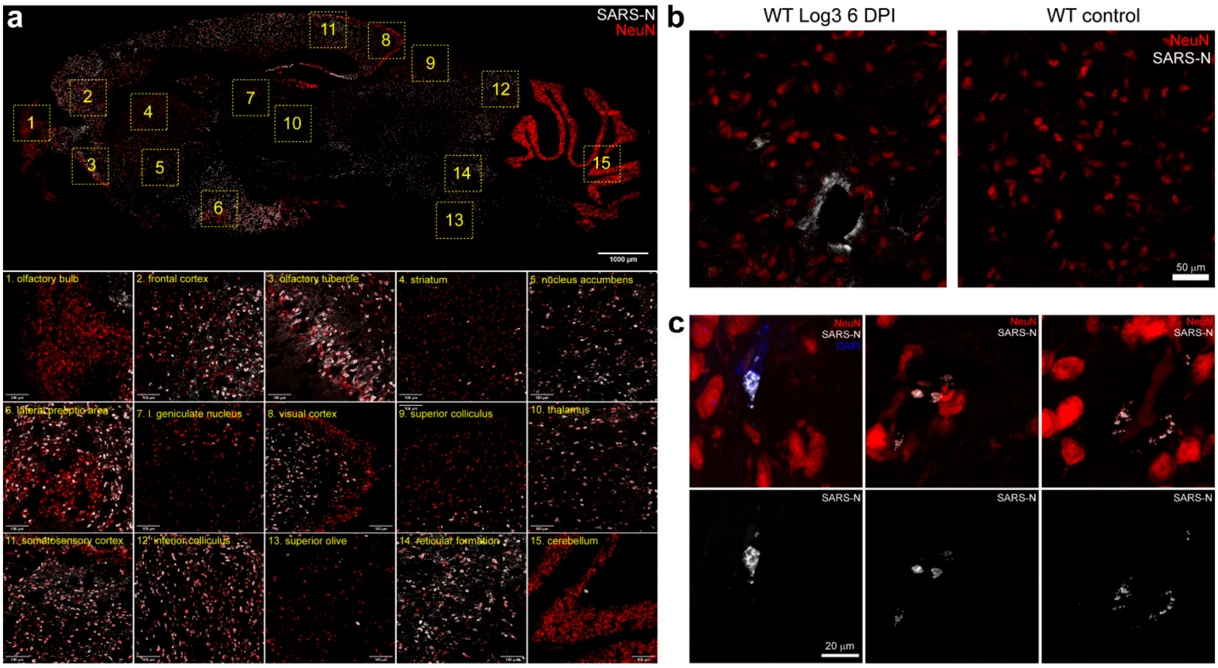

Supplementary Figure S4. Immunofluorescence for SARS-N protein and NeuN in sections of brains taken from hACE2 mice, WT mice, and golden Syrian hamsters. All images were acquired using a Leica SP8 confocal microscope, using identical image acquisition settings (laser power and gain) across all sections from each tissue type shown. All images were colorized, z-projected, and prepared using identical contrast and brightness parameters in ImageJ. (**a**), SARS-N+ neurons were observed throughout multiple brain regions with some notable exceptions. Few SARS-N+ cells were detected in the granule cell layer of the olfactory bulb, the medium spiny neurons in the striatum, and the granule cells in the cerebellum. Relatively few SARS-N+ neurons were identified in the lateral geniculate nucleus, layers 2 and 3 of the visual cortex, and in the superior colliculus. This stood in contrast to adjacent non-visual areas (thalamus, somatosensory cortex, inferior colliculus), which had numerous SARS-N+ neurons. The fact that some of these areas contain GABAergic neurons suggests that inhibitory neurons may be spared infection, at least at this time point. See Figure S3 for control hACE2 brain sections. (**b**), SARS-N is present in a minority of neurons in infected WT mice. Of note, heavy SARS-N staining can be seen in a circular ring of tissue, possibly vasculature, with scattered SARS-N signal throughout the surrounding area. Minimal background immunofluorescence is observed in sections from uninfected mice. It is worth noting that while SARS-N was minimally detected by immunostaining in the brains of WT mice, as reported by other groups, SARS-CoV-2 RNA was readily detected by RT-qPCR in discrete brain regions at 3 and 6 dpi. Also, infectious virus was recovered via plaque assay from the hippocampi (3 log group: 2 PFU/mg homogenate; 5 log group: 5 PFU/mg homogenate) and brainstem (3 PFU/mg homogenate) of WT mice at 6 dpi. (**c**)**,** SARS-N is present in some cells in infected hamster brains, which appear to be associated with vasculature. See Video S5 for 3D rendering of this image. The use of multiple complimentary assays when applied to discrete brain regions increases the likelihood of detecting neuroinvasion in these mice. See Figure S6 for additional antibody validation via western blot.

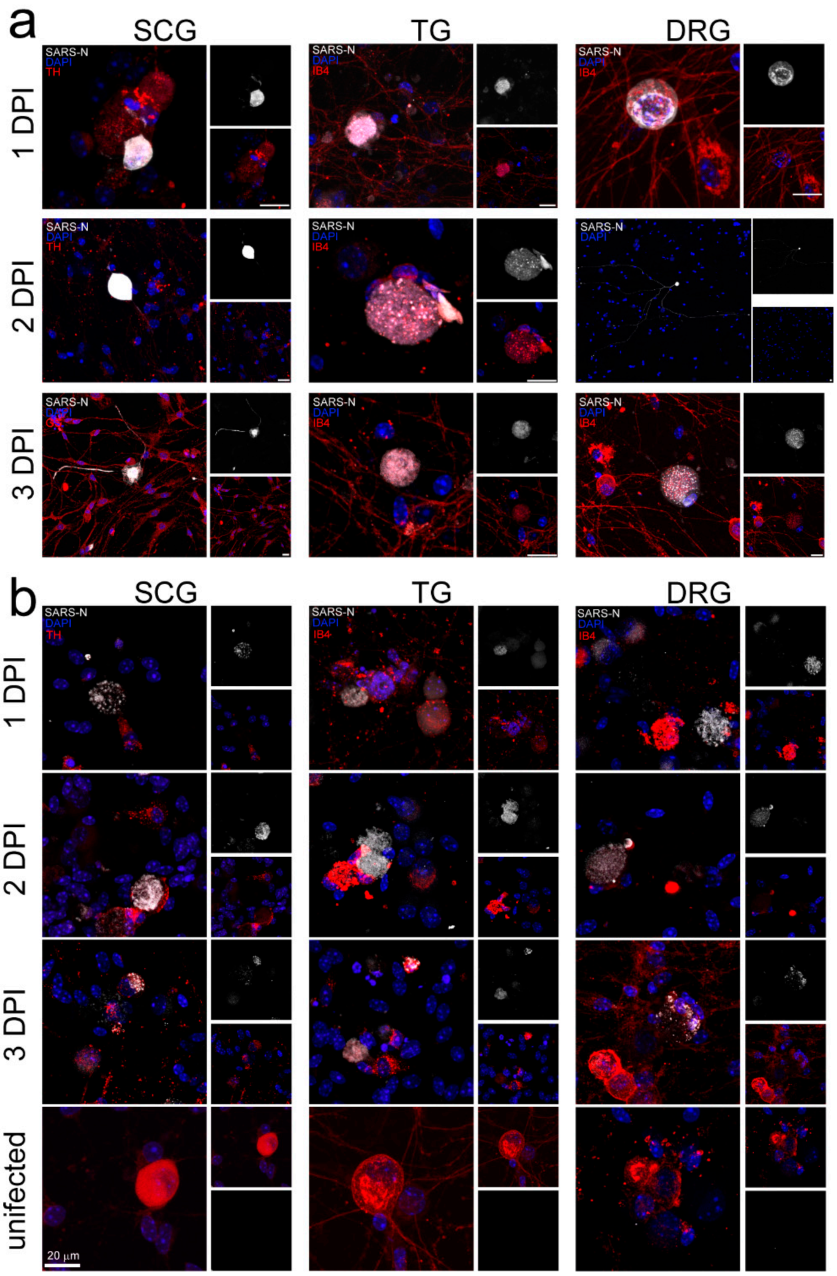

Supplementary Figure S5. Immunofluorescence for SARS-N protein in *in vitro* cells taken from hACE2 and WT peripheral ganglion cultures. Cells were fixed 1-3 dpi and stained for SARS-N and various counterstains. All images were acquired using a Leica SP8 confocal microscope. Because of substantial variability in intensity of SARS-N immunofluorescence, laser power and gain were adjusted in order to highlight features of each cell. (**a**), Day 2 LS-DRG is a montage image to show SARS-N detected throughout the neurites of one infected neuron; only DAPI is shown. SARS-N is present in neurons in the SCG, TG, and LS-DRG in infected hACE2 mice. (**b**), Punctate SARS-N immunoreactivity is visible in many infected cells in WT mice. Virtually no background is visible in uninfected cells. Identical imaging and post-processing parameters were used for all images. TH = tyrosine hydroxylase, IB4 = Isolectin-B4, GS = glutamine synthetase, SARS-N = SARS-CoV-2 nucleocapsid, DAPI = 4′,6-diamidino-2-phenylindole. Scale bar = 20 µm. See Figure S6 for additional antibody validation via western blot, hACE2 genotyping, and hACE2 protein expression.

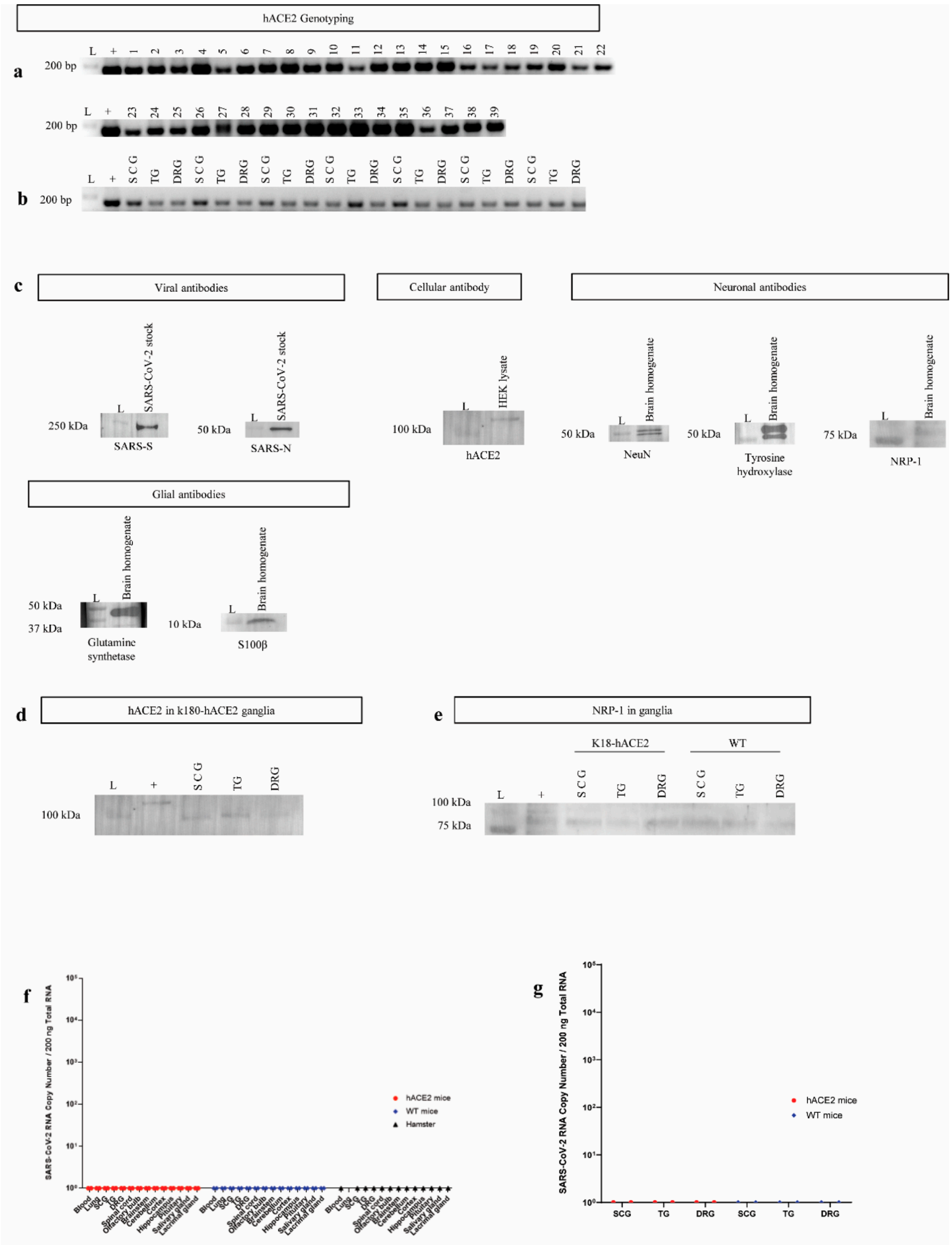

Supplementary Figure S6. **Validation of hACE2 mice, immunostaining antibodies, and SARS-CoV-2 N1 specific RT-qPCR assay.** (**a**), hACE2 mice (n=39; 35 infected, 4 controls) were genotyped following Jackson Laboratory protocol #38170 V2. The presence of a PCR product at ≈170 bp, the predicted size of the hACE2 amplicon, confirms presence of the transgene in these mice. (**b**), SCGs, TGs, LS-DRGs used for neuronal culture infection studies (n=7 independent cultures) were genotyped as above to confirm presence of the transgene. The presence of an amplicon at ≈170 bp confirms presence of the transgene in these primary neuronal cultures. (**c**), Antibodies used in immunostaining were validated by western blot using positive controls recommended by their manufacturers. (**d**),

Expression of hACE2 protein was confirmed via western blot in homogenates of SCGs, TGs, and LS-DRGs from hACE2 mice by observation of a band at ≈100 kDa (molecular weight 100-110 kDa). A mild elevation of ACE2 in the positive control (HEK293 cell homogenate) above 100 kDa is observed but is within the range of the molecular weight. It is not uncommon for ACE2 in HEK293 cell homogenate to appear mildly elevated above 100 kDa on western blots likely due to modifications in kidney epithelial cells that are not present in ganglia (R&D Systems). (**e**), Expression for NRP-1 protein was confirmed via western blot in homogenates of SCGs, TGs, and LS-DRGs from hACE2 and WT mice. (**f**), The SARS-CoV-2 N1 specific RT-qPCR assay was validated for each tissue assessed in hACE2 mice, WT mice, and hamsters by assessing negative control tissues from uninfected hACE2 mice (n=2), WT mice (n=2), and hamsters (n=1). (**g**), The SARS-CoV-2 N1 specific RT-qPCR assay was validated for neuronal culture infection studies by assessing negative control uninfected cultures of SCGs, TGs, and LS-DRGs from hACE2 and WT mice (n=2 per ganglia per mouse type).
